# Public health journals’ requirements for authors to disclose funding and conflicts of interest: a cross-sectional study

**DOI:** 10.1186/s12889-018-5456-z

**Published:** 2018-04-23

**Authors:** Karim N. Daou, Maram B. Hakoum, Assem M. Khamis, Lama Bou-Karroum, Ahmed Ali, Joseph R. Habib, Aline T. Semaan, Gordon Guyatt, Elie A. Akl

**Affiliations:** 1 Department of Epidemiology and Population Health, Faculty of Health Sciences, American University of Beirut, Beirut, Lebanon; 2 Clinical Research Institute, American University of Beirut Medical Center, Beirut, Lebanon; 3 Center for Systematic Reviews for Health Policy and Systems Research, American University of Beirut, Beirut, Lebanon; 4 Faculty of Health Sciences, American University of Beirut, Beirut, Lebanon; 5 Faculty of Medicine, American University of Beirut, Beirut, Lebanon; 6 Department of Health Research Methods, Evidence, and Impact, McMaster University, Hamilton, Ontario Canada; 7 Department of Internal Medicine, American University of Beirut, Beirut, Lebanon

**Keywords:** Conflict of interest, Funding, Public health, Journal policy

## Abstract

**Background:**

Public health journals need to have clear policies for reporting the funding of studies and authors’ personal financial and non-financial conflicts of interest (COI) disclosures. This study aims to assess the policies of public health journals on reporting of study funding and the disclosure of authors’ COIs.

**Methods:**

This is a cross-sectional study of “Public, Environmental & Occupational Health” journals. Teams of two researchers abstracted data in duplicate and independently using REDCap software.

**Results:**

Of 173 public health journals, 155 (90%) had a policy for reporting study funding information. Out of these, a majority did not require reporting of the phase of the study for which funding was received (88%), nor the types of funding sources (87%). Of the 173 journals, 163 (94%) had a policy requiring disclosure of authors’ COI. However, the majority of these journals did not require financial conflicts of interest disclosures relating to institutions (75%) nor to the author’s family members (90%) while 56% required the disclosure of at least one form of non-financial COI.

**Conclusions:**

The policies of the majority of public health journals do not require the reporting of important details such as the role of the funder, and non-financial COI. Journals and publishers should consider revising their editorial policies to ensure complete and transparent reporting of funding and COI.

## Background

Conflicts of interest (COI) are ‘circumstances that create a risk that professional judgments or actions regarding a primary interest will be unduly influenced by a secondary interest’, as defined by the Institute of Medicine [1].

Conflicts of interests are common in public health research [2]. Bekelman et al. found that one fourth of investigators in biomedical research are associated with industry, and two thirds of academic establishments hold capital shares in start-ups that fund research performed at these same institutions [3]. Researchers conducting Medical Research Council (MRC) projects have received funding from organizations including Nestlé, the Institute of Brewing and Distilling, Weight Watchers International, NutriLicious, Sainsbury’s, W K Kellogg Institute, and GlaxoSmithKline [4].

There have been doubts about how the major industries of relevance to public health have funded research work related to their products. An analysis of internal industry documents suggested that the sugar industry sponsored in the 1960s and 1970s several research programs through the Sugar Research Foundations that casted doubts about the harms of sucrose in developing cardiovascular diseases [5]. There was no disclosure of funding, grants or roles [5]. Similar research by Barnes and Bero suggested that the tobacco industry funded “special-reviewed projects” through the Center for Indoor Air Research (CIAR) to develop scientific data that “it can use in legislative and legal settings”. The investigators also concluded that CIAR may have funded peer-reviewed projects to enhance the credibility of the tobacco industry and divert attention from environmental tobacco smoke as an indoor air pollutant [6]. Also, there is evidence that the alcohol industry used a similar approach through the Center for Alcohol Policies [7].

Indeed, industry funding of research is likely to bias its findings in favor of the industry. Ahn et al. found that financial ties between principal investigators and the pharmaceutical industry was associated with positive study outcomes (OR = 3.37) [8]. Lesser et al. found that articles with all industry funding, compared with those with no industry funding, were associated with increased odds of favorable conclusions (OR = 7.61) [9].

There is evidence that industry has been able to negatively affect public health policies, e.g., in the area of non-communicable diseases [10]. Between 2011 and 2015, Coco-Cola and PepsiCo sponsored 95 medical and public health institutions [11]. Concomitantly, these two largest soda companies were able to impede 29 public health bills projected to lower sugar consumption [11]. This negative impact is at least partially mediated by the COIs of members of policy making bodies [12].

A methodological survey conducted in 2016 found that authors of 49% and 33% of published Cochrane and non-Cochrane systematic reviews reported COIs respectively, and reported individual and financial COIs more frequently than institutional and non-financial COIs [13]. In meta-analysis on antihypertensive drugs, financial ties to drug companies were associated with favorable conclusions (OR = 4.09) [14].

Although most discussions on COI disclosure have focused on financial relationships [15], there is increasing interest in non-financial COIs such as professional, intellectual and personal conflicts [16]. Half of cases presented to Committee on Publication Ethics (COPE) forum for advice involved non-financial COIs [17]. Further, it is possible for editors to have conflicts that could affect their decisions to accept or reject submitted manuscripts [18].

Unreported COIs by authors and the lack of declaration of funding from industry to institutions and investigators may be associated with bias in the findings of research conducted for public health purposes. Thus, it is important to assess the policies of public health journals for the disclosure of COI and funding. The two objectives of the study were to assess the policies of public health journals regarding the reporting of funding information and to assess the policies regarding the disclosure by authors of any financial and non-financial COI.

## Methods

### Definitions and overall design

For this study, we adopted the Institute of Medicine’s definition of COI, provided above [1]. We defined a COI policy as one that required COI disclosure by at least one of the authors. We used a cross-sectional design to review the journals’ instructions for authors on disclosing COI and funding.

### Eligibility criteria

The study focused on journals categorized as “Public, Environmental & Occupational Health”, in Social Science Citation Index (SCIE) edition of the Journal Citation Reports (JCR) of the Web of Science database (https://jcr.incites.thomsonreuters.com). We included in our study all of the 173 journals listed in the SCIE in January 2017. We did not limit our selection by language of publication.

### Data collection process

We used three sources to abstract relevant data:

- Instructions for authors on the journal website. This information is publically available and not password protected;
- Instructions in the journal online submission system. Given access to the online system is typically password protected, one of the investigators (KND) registered with and logged in to access the relevant information;
- Publisher website when redirected from the journal website or the online submission system.

**Fig. 1:**
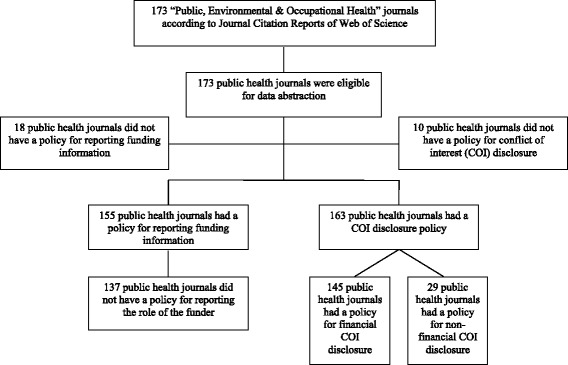
Flow diagram for selection of journals

Three teams of two reviewers collected data in a duplicate and independent manner using a standardized and pilot tested data abstraction form (Fig. 1). They compared results and resolved disagreements through discussion or with the help of a third reviewer (MBH or EAA) when needed. All reviewers conducted calibration exercises prior to data collection. We collected and managed data using the Research Electronic Data Capture (REDCap) tools hosted at the American University of Beirut [19]. REDCap is a secure, web-based application for supporting data capture for research studies.

### Data collected

We collected data regarding the characteristics of the journals, the COI disclosure policies, and funding policies (the processes were limited to explicit journal policies):

*General characteristics of the journal:*

- Journal Impact factor (according to the latest JCR Science Edition);
- Journal Category, other than ‘Public, Environmental & Occupational Health’ (according to JCR Science Edition 2015);
- International Committee of Medical Journal Editors (ICMJE) membership (according to ICMJE website);
- Journal affiliation with a professional organization (according to NCBI or journal website);
- Journal publisher (according to NCBI or journal website);
- Committee on Publication Ethics (COPE) membership (according to COPE website);
- Author’s requirement to comply with reporting statements (e.g., CONSORT for trials).

*Funding reporting policy:*

- Characteristics of the journal policy on reporting of funding information
- Elements of the funding information required by the policy (e.g., source, amount)
- Whether the role of funder is required by the policy (e.g., approval of publication)

*COI disclosure policy:*

- Presence and type of disclosure policy (i.e., the journal’s policy or the publisher’s policy);
- Requirement of COI policy for individuals/groups to disclose COI;
- Presence of a form for COI disclosure;
- Disclosure requirement as part of the manuscript submission;
- Policy description of whether and how COI disclosure will affect editorial process;
- Policy description of how non-disclosure may impact the editorial process;
- Policy description of procedures to verify authors’ COI disclosures.

*Journal requirements for financial COI disclosure:*

- Policy requirement of disclosure of financial relationships (whether of the author, author’s family members, or author’s institution);
- Policy specification of types of financial relationships to be disclosed;
- Disclosure of sources and amount of payment;
- Disclosure of duration of the financial relationship.

*Journal requirements for non-financial COI disclosure:*

- Policy requirement of disclosure of non-financial COIs;
- Policy specification of types of non-financial relationships to be disclosed.

### Data analysis

We checked for any missing data or erroneous entries. For the descriptive analyses of journals’ characteristics, we used frequencies and percentages for categorical variables. We conducted regression analyses to identify relationships between [1] existence of a funding disclosure policy, and explicit requirement for disclosure of COI and the following independent variables: COPE, ICMJE and impact factor. None of these analyses was significant. We conducted analysis using SPSS version 23.0 (SPSS INC., Chicago, IL, USA).

## Results

We included the 173 journals listed by the Web of Science under “Public, Occupational and Environmental Health” category. Figure 1 presents the flow diagram.

### General characteristics of the journals

Table 1 provides the results of the general characteristics of the 173 included journals. Twenty-one percent of those journals were members of the ICMJE, 67% were members of COPE, and 75% were affiliated to a professional organization. The most common publisher was Elsevier (15%) associated with public health journals.

**Table 1:** General characteristics of the journals (*N* = 173)

	n (%)
Impact factor [median (IQR)]	1.63 (1.13–2.55)
Listed under a category in addition to “Public, Environmental and Occupational Health” category	92 (53)
*Environmental Sciences*	20 (12)
*Health Care Sciences and Services*	15 (9)
*Infectious Diseases*	10 (6)
Publisher	
*Elsevier*	25 (15)
*Taylor & Francis*	18 (10)
*Springer*	14 (8)
*Wiley-Blackwell*	14 (8)
*Oxford University Press*	12 (7)
*Bio Med Central*	9 (5)
*Sage*	8 (5)
*Cambridge University Press*	6 (4)
*BMJ*	4 (2)
*Mary Ann Liebert*	4 (2)
*Lippincott Williams & Wilkins*	3 (2)
*Other*	56 (32)
Member of the ICMJE ^a^	37 (21)
Affiliated with a professional organization	129 (75)
Member of the Committee on Publication Ethics (COPE)	115 (67)
Requires compliance with at least one of the reporting statements	67 (39)

^a^*ICMJE* International Committee of Medical Journal Editors

### General characteristics of the journals’ policies on reporting of information regarding funding of the research being reported

Out of the 173 included journals, 155 (90%) had a policy regarding the reporting of information regarding funding of the research. Table 2 summarizes the characteristics of those policies. Ninety-two percent of the policies were specific to the journal. No policy explicitly included procedures to deal with non-reporting or under-reporting of funding. The most frequent two methods for reporting of funding information were online as part of the submission process (74%) and in the body of the manuscript (63%).

**Table 2:** General characteristics of the journals’ policies on reporting of funding information (*N* = 155)

	n (%)
Policy is specific to:	
*The journal*	143 (92)
*The publisher*	5 (3)
*Both*	7 (5)
Reported procedures on dealing with non-reporting or under-reporting of funding	0 (0)
Submission form of funding information	
*Online as part of the submission process*	114 (74)
*In the body of the manuscript*	97 (63)
*In a file separate from the manuscript*	24 (16)
*In a free-standing form*	7 (5)
*Not specified*	6 (4)
Distinguished between “funder” and “sponsor”^a^	18 (12)

^a^Sponsor: an individual, academic institution, company or governmental agency that takes responsibility for and initiates a clinical investigation. Funder: an individual or organization that provides money for a study

### Elements of the funding information required by the policies

Table 3 provides the elements of funding required by the policies of the 155 journals requiring the reporting of funding information. Respectively, 88% and 87% of policies did not require the reporting of the phase(s) of the research to which the funding applied, or the types of funding sources. Twelve percent of policies required the disclosure of whether a not-for-profit funding source is supported by a for-profit entity. Forty-three percent of policies did not specify the types of funding to be reported. Monetary support was the most frequent type of funding the policies required to be reported (53%). However, none of these required the reporting of the amount of monetary support.

**Table 3:** Elements of the funding information required by the policies (N = 155)

	n (%)
Phase(s) of the research study for which reporting of funding is required	
*Not specified*	136 (88)
*Conduct*	18 (12)
*Reporting*	13 (8)
*Planning*	3 (2)
*Other*	0 (0)
Specification of types of funding sources	
*Not specified*	134 (87)
*Government*	15 (10)
*Private-for-profit*	10 (7)
*Private not-for-profit*	10 (7)
*Internal funding*	1 (1)
*Inter-government*	0 (0)
*Other*	1 (1)
Required the disclosure of whether a not-for-profit funding source is supported by a for-profit entity	18 (12)
Types of funding to be reported	
*Monetary support*	82 (53)
*Provision of supplies*	15 (10)
*Assistance in manuscript writing*	4 (3)
*Other*	4 (3)
*Not specified*	66 (43)
Amount of monetary support to be reported (*N* = 82)	
*Not required*	82 (100)
*Yes, irrespective of amount*	0 (0)
*Yes, for amount above a specific value*	0 (0)
Additional information (if policy requirement of reporting on provision of supplies) (N = 15)	
*Not specified*	9 (60)
*Types of supplies*	6 (40)
*Monetary value of supplies*	0 (0)
*Other*	0 (0)

### Characteristics of the information required by the policies on the role(s) of funder

**Fig. 2:**
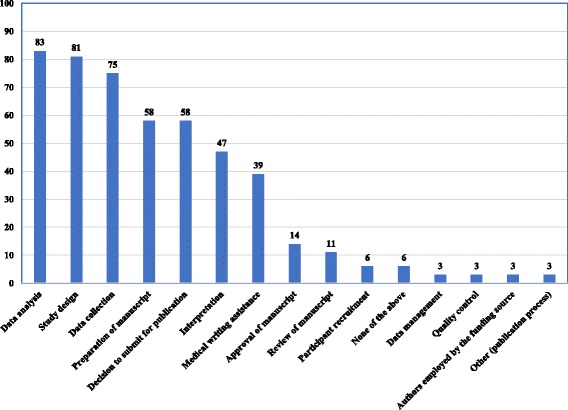
Percentage of policies requiring the reporting of the role of funder for the different steps of the research (*N* = 36)

Out of the 155 journal policies for reporting of funding information, 36 (23%) required reporting on the role(s) of funder. Figure 2 shows the percentages of policies requiring the disclosure of the role of the funder for each of the steps of the research process. The most frequently required steps were data analysis, study design, and data collection (83%, 81% and 75% respectively). Four policies clearly requested authors to report on having independent and full control in at least one of the steps of the research process: study design (*n* = 1), data collection (n = 1), data management (*n* = 2), data analysis (n = 1), decision to submit for publication (n = 2), access to the data (n = 1), and freedom to conduct the research (n = 1) (Table 4).

**Table 4:** Policies on authors to report on having independent and full control in at least one of the steps of the research process (*N* = 4)

Independent and full control in any step of the research study (N = 4)	n (%)
*Design*	1 (25)
*Data collection*	1 (25)
*Data management*	2 (50)
*Data analysis*	1 (25)
*Quality control*	0 (0)
*Preparation of manuscript*	0 (0)
*Review of manuscript*	0 (0)
*Approval of manuscript*	0 (0)
*Decision to submit for publication*	2 (50)
*Access to the data*	1 (25)
*Freedom to conduct the research*	1 (25)
*None of the above*	0 (0)

### Characteristics of the COI disclosure policies

Out of the 173 eligible journals, 163 (94%) had a disclosure policy regarding individual authors’ COI. Table 5 summarizes the characteristics of those policies. Seventy-seven percent of the policies were specific to the journals while 18% were specific to both the publishers and the journals. The majority of policies required the disclosure of COI at the time of manuscript submission (98%), in a form of narrative statement (91%), during the online submission process (59%), or in the body of the manuscript (58%). COI disclosures were stated to be made available to the public within the published manuscript in 50% of policies. Seven percent of the policies mentioned the possibility of verifying COI disclosures following revision or acceptance. While 11% of journals would contact authors for more details on disclosed COI, 19% of policies describe the effect of disclosures on the editorial process (e.g., editors and reviewers may decide to disqualify review from publication or reject the manuscript); and 10% clearly mention a potential impact of not disclosing COI on the editorial process (e.g., editors may correct, reject or retract the publication). No policy described procedures to verify authors’ COI disclosures.

**Table 5:** Characteristics of conflict of interest (COI) disclosure policy (*N* = 163)

	n (%)
COI disclosure policy	
*Specific to the journal*	125 (77)
*Specific to the publisher*	8 (5)
*Specific to both*	30 (18)
Timing of COI disclosure	
*At the time of manuscript submission*	159 (98)
*At the time of manuscript acceptance*	9 (6)
*Unclear*	3 (2)
Form of COI disclosure	
*A narrative statement*	149 (91)
*ICMJE*^a^ *Uniform Disclosure Form*	24 (15)
*Journal form*	24 (15)
*Publisher form*	9 (6)
*Modified ICMJE form*	6 (4)
*A COI disclosure form different from the ICMJE Uniform Disclosure Form*	2 (1)
Location of COI disclosure	
*Online as part of the submission process*	96 (59)
*In the body of the manuscript*	95 (58)
*In a file separate from the manuscript (*e.g.*, cover letter)*	61 (37)
Via *email sent by authors to the journal*	2 (1)
*Unclear*	8 (5)
Making of COI disclosures publically available	
*Not clear*	82 (50)
*Within the published manuscript*	81 (50)
*In an online form*	0 (0)
*Upon request*	0 (0)
*Explicitly states that disclosures will not be made available*	0 (0)
Requested COI disclosure related to	
*Submitted work*	137 (84)
*Work not related to the submitted work (or outside the submitted work)*	7 (4)
*Not specified*	26 (16)
Effect of COI disclosures on the editorial process described	31 (19)
Authors may be contacted for more details on disclosed COI	18 (11)
COI disclosure may be verified following revision or acceptance	11 (7)
Procedures to verify authors’ COI disclosures described	0 (0)
Effect of not disclosing COI on editorial process described	16 (10)

^a^ *ICMJE* International Committee of Medical Journal Editors

### Requirements for financial COI disclosure

Table 6 describes the requirements for financial COI disclosure of the 163 journals with a COI disclosure policy. Out of journals having COI disclosure policies, 25% and 10% respectively require the disclosure of the financial relationships of authors’ institutions and family members. The most commonly required forms of financial COI are as stock ownership (51%), grant/research support (45%), direct employment (45%) and serving as an advisor, consultant, or public advocate (43%). Eighty-seven percent of the policies do not specify the duration for which to disclose the financial COI. Among those in which it was necessary for the previous number of months to be reported (2%), 90% specified a duration of 36 months. Although 39% of journals’ policies explicitly required reporting the source of financial benefit, only one required reporting of the monetary value.

**Table 6:** Journal policy requirements for financial conflict of interest (COI) disclosure (N = 163)

	n (%)
Disclosure of financial relationships	145 (89%)
*Of author(s)*	145 (89)
*Of author’s institution*	40 (25)
*Of author’s family member(s)*	17 (10)
*Not explicitly required*	18 (11)
*Of other*	1 (0)
Types of financial disclosure	
*Stock ownership*	83 (51)
*Grant/research support*	73 (45)
*Direct employment*	73 (45)
*Serving as an advisor, consultant, or public advocate*	70 (43)
*Honoraria for speaking, writing, or reviewing on the topic discussed in the manuscript*	53 (33)
*Personal fees*	50 (31)
*Indirect financial support*	47 (29)
*Speaker bureaus or board membership*	37 (23)
*Royalties*	26 (16)
*Other type of financial COI*	4 (3)
*Required but did not specify which types of financial COI*	40 (25)
*Not required*	28 (17)
Duration for which to disclose financial COI	
*Not specified*	142 (87)
*Yes, for the previous number of months*	20 (12)
*Yes, irrespective of timing (*i.e.*, at any time)*	1 (1)
Number of months of required duration (*N* = 20)	
*Within 12 months*	0 (0)
*12 months*	0 (0)
*24 months*	1 (5)
*36 months*	18 (90)
*4 years*	0 (0)
*5 years*	1 (5)
*> 5 years*	0 (0)
Time frame for financial COI disclosure	
*Time of the submission*	11 (7)
*At the initiation of the study*	5 (3)
*Not specified*	4 (3)
Required disclosure of financial COI in the near future	12 (7)
Required duration of financial COI outside submitted work	1 (0)
Explicitly required source of financial benefit	64 (39)
Required monetary value of financial benefit	
*Not required*	162 (100)
*Yes, irrespective of amount*	1 (0)
*Yes, for amounts above a specific value*	0 (0)
Required disclosure of any patents	63 (41)

### Requirements for non-financial COI disclosure

Ninety-two of the 163 COI journals’ policies (56%) required the disclosure of at least one form of non-financial COI. The top 4 descriptors of non-financial COI were: “personal relationship” (33%), “personal” (20%), “academic competition” and “non-financial COI” (14%) (Table 7).

**Table 7:** Journal policy requirements for non-financial conflict of interest (COI) disclosure (N = 163)

	n (%)
Required disclosure of the following non-financial COI in the “exact wording” (*N* = 92)	92 (56)
*Not required by the policy*	71 (44)
*“ Personal relationship ”*	54 (33)
*“ Personal ”*	33 (20)
*“ Academic competition ”*	22 (14)
*“ Non-Financial COI ”*	22 (14)
*“ Political ”*	21 (13)
*“ Professional ”*	20 (12)
*“ Academic ”*	16 (10)
*“ Intellectual ”*	13 (8)
*“ Ideological ”*	11 (7)
*“ Religious views ”*	11 (7)
*“ Institutional ”*	10 (6)
*“ Intellectual passion ”*	7 (4)
*“ Personal opinion / belief ”*	7 (4)
*“Other”*	6 (4)

## Discussion

### Summary of findings

Of 173 public health journals, 155 (90%) had a policy for reporting study funding information. Out of these, a majority did not require reporting of the phase of the study for which funding was received (88%), nor the types of funding sources (87%). Of the 173 journals, 163 (94%) had a policy requiring disclosure of authors’ COI. However, the majority of these journals did not require financial conflicts of interest disclosures relating to institutions (75%) nor to the author’s family members (90%) while 56% required the disclosure of at least one form of non-financial COI.

### Strengths and limitations

This is the first study to investigate, using systematic methodology, public health journal policies for reporting of funding information. Our study assessed this topic in a detailed way (e.g., potential effect of disclosures and incomplete or imprecise COI disclosures on the editorial process). One limitation is that we might have missed funding and COI policies that journals implemented during later steps of the editorial process, especially that we did not contact the journals.

### Comparison to similar studies

Shawwa et al. reported that 99% of Core Clinical Journals had a policy on COI disclosure in comparison to 94% of public health journals [15]. Clinical journals were more likely to require specifying financial and non-financial COIs (100% and 57% respectively) compared to public health journals (89% and 18% respectively) [20]. Also, a higher percentage of clinical journals, compared to public health journals, specified measures to deal with inaccurate or incomplete disclosures statements (23% vs. 10%) [20]. Furthermore, no public health journal reported procedures to verify the accuracy or completeness of authors’ disclosed COIs, compared to 17% of clinical journals [20].

### Implications for editorial policy

The issues of conflicts and their potential influence on what articles are published, their results, and the framing of the conclusions, suggest that editors and publishers of public health journals should reevaluate their policies regarding reporting of funding and disclosure of authors’ COI. Such a reevaluation should be based on the principles related to honesty and objectivity of the editorial process [21].

A comprehensive policy for reporting funding should require the specification of the phase of research study to which funding applied (planning, conduct or reporting), the specific type of funding source (governmental or not, for profit or not) as well as the type of funding (monetary support, provision of supplies, assistance in writing) and its amount.

While improvement in editorial policies can include the verification of financial COI of authors’ institutions and their family members, findings in Table 7 also suggest that policies are limited in their disclosure of non-financial COIs. These might include intellectual COI related to authorship, academic affiliations, institutional, personnel or professional COIs, political and religious affiliations.

### Implications for research

Ideally, journals would implement a systematic approach with acceptable validity and reliability that compels authors to reveal financial and non-financial interests underlying their research work. Although a significant fraction of public health journals included policies offering considerable description on the study’s funding and authors’ own COIs, COIs related to their family members and affiliated departments are seldom addressed. There is a need for well-defined operational definitions to simplify both the disclosure of COI and funding information by authors and their verification assessment and control by the editorial board. Future research should also emphasize credible evidence for the interventions and guidelines to manage funding information and COI.

## Conclusion

Our findings have demonstrated that most of the journals requested the disclosure of information regarding study funding, and financial and non-financial COIs of study authors. However, the majority failed to require important details such as the role of the funder and the impact of inaccurate or incomplete COI disclosure on the editorial process. From a more general perspective, undeclared COIs by authors and the underreporting of funding from companies to organizations and investigators could be a cause of biased findings conducted for public health purposes.

## Abbreviations

COI: Conflicts of interest
COPE: Committee on Publication Ethics
ICMJE: International Committee of Medical Journal Editors
JCR: Journal Citation Reports
REDCap: Research Electronic Data Capture
SCIE: Social Science Citation Index
